# Burr Detection and Edge Condition in Ceramic Brushing of Glass and Carbon Fabric-Reinforced Composites Using Recurrence Analysis of Force Signals: A Feasibility Study

**DOI:** 10.3390/ma19214763

**Published:** 2026-11-09

**Authors:** Jakub Matuszak, Krzysztof Ciecieląg

**Affiliations:** Department of Production Engineering, Mechanical Engineering Faculty, Lublin University of Technology, Nadbystrzycka 36, 20-618 Lublin, Poland; k.ciecielag@pollub.pl

**Keywords:** brushing, deburring, burr detection, GFRP, CFRP, recurrence analysis, force signal, edge condition, ceramic brushes

## Abstract

**Highlights:**

- The burr-free edge was represented by milled chamfered segments 0.1–0.4 mm deep, alternating with as-milled segments with burrs.
- The force signal recorded during brushing was analysed using recurrence plots (RP) and recurrence quantification analysis (RQA) measures.
- Under the tested conditions, chamfered segments ≥ 0.2 mm deep were distinguishable from the as-milled edge with burrs; 0.1 mm segments in most cases were not.
- Chamfer width increased with fibre stiffness, from 389 to 1040 µm.

**Abstract:**

Burrs formed during the machining of fibre-reinforced polymer composites reduce safety and product quality and are usually inspected off-process. This study assessed whether as-milled edge segments with burrs can be distinguished from burr-free chamfered segments on the basis of the force signal recorded during edge brushing, as a first step towards in-process burr detection, and how brush fibre stiffness affects the edge condition. On one edge of glass (GFRP) and carbon (CFRP) fabric-reinforced specimens, positioned at 45° to the tool axis, chamfered segments 0.1–0.4 mm deep, representing a burr-free edge, alternated with as-milled segments with burrs. The edges were brushed with four ceramic-fibre brushes of increasing stiffness (S1–S4) at constant parameters, and the force signal was analysed using recurrence plots (RP) and recurrence quantification analysis (RQA). Chamfers 0.1 mm deep did not produce unambiguous changes in the recurrence structure. For the stiff brushes (S3, S4), chamfers 0.2–0.4 mm deep produced local RP densifications consistent with their location. In the RQA, for chamfers ≥ 0.2 mm, DET (determinism) and L (average diagonal line length) mostly rose above and LAM(laminarity) and TT (trapping time) fell below the burred-edge reference levels. The chamfer width produced by brushing increased with fibre stiffness, from 588 to 1040 µm (GFRP) and from 389 to 1005 µm (CFRP). Under the tested conditions, the force signal distinguished the two edge conditions; its use for detecting burrs themselves requires further validation on edges with quantified burrs.

## 1. Introduction

Glass (GFRP) and carbon (CFRP) fibre-reinforced polymer composites are a primary material for lightweight structures in the aerospace, automotive, wind energy and sports industries owing to their high specific strength and stiffness and their corrosion resistance [1]. Composite components are usually manufactured by near-net-shape methods, most often from prepregs cured in an autoclave, yet almost every part subsequently requires machining: edge trimming to remove the technological allowance, milling of contours and assembly areas, and drilling of holes for riveted and bolted joints [2,3,4]. Matos et al. [5] point out that although CFRP components are produced to near-net shape, increasingly stringent dimensional tolerance requirements in the aerospace and automotive industries mean that edge trimming remains the principal finishing operation. Its purpose is to remove the material remaining after manufacturing and to give the edge the required micro- and macrogeometry. The authors investigated the edge trimming of CFRP laminates with laser-sharpened, thick-film diamond-coated carbide end mills. They showed that cutting forces and edge quality depend primarily on the fibre cutting angle (the angle between the fibre direction and the cutting direction), and that cross-ply laminates achieved lower roughness than unidirectional ones.

Machining composites differs fundamentally from machining metals. The material is heterogeneous and anisotropic, the fibres (especially glass and carbon) are highly abrasive, and the polymer matrix has low thermal conductivity and limited temperature resistance. The material separation mechanism depends on the angle between the cutting edge and the fibre direction and consists of brittle fibre fracture and matrix shearing rather than plastic flow, as in metals [6,7]. Liu et al. [8] showed that the fracture mechanism of CFRP during milling is governed by the fibre cutting angle. At 0°, interface separation between fibre and matrix dominates; up to 90°, interface separation and shear fracture occur; and above 90°, the mechanism rapidly changes to bending fracture. Bending fracture sharply deteriorates surface quality and promotes the propagation of damage into the material. Each ply of a multidirectional laminate retains the fracture mechanism of a unidirectional laminate. In the unsupported outer ply, burrs are formed at angles below 90°, and delamination above 90°.

The consequences of this mechanism are characteristic machining defects: delamination of the entry and exit plies, fibre fraying, uncut fibres, fibre pull-out, matrix cracking and thermal degradation of the matrix [9,10,11,12]. In metals, a burr is a plastically deformed portion of material that has not separated as a chip [13,14]. In fabric composites, the term “burr” most often refers to bundles of uncut fibres and fragments of the outer ply protruding beyond the theoretical edge contour; they occur mainly in the outer plies, which lack the support of adjacent plies. Can [15] showed that the type of edge damage in CFRP after edge trimming depends on the milling strategy. Peripheral milling with the tool perpendicular to the workpiece produced long uncut fibres protruding from the edge, while a tilted tool produced shorter protruding fibres and plies broken inwards into the material. Roughness and damage size decreased with increasing number of flutes and cutting speed and with decreasing feed per tooth. Li et al. [16], slot milling cross-ply (0°/90° and 45°/135°) and fabric laminates, observed burrs at the entry edge consisting of fibres up to approx. 5.6 mm long. The length and number of these burrs depended strongly on fibre orientation and cutting angle, and the defects were located mainly in the 45°/135° plies. Machining parameters and strategy can therefore reduce the number of uncut fibres but cannot eliminate them completely, which justifies a separate deburring operation.

Edge burrs are both a quality problem and a matter of product safety in service. According to the review by Geier et al. [17], burrs and delamination formed during edge trimming are the most serious edge defects in CFRP. They reduce the strength of the component and affect damage initiation and propagation, and burrs additionally impair assembly accuracy. Therefore, neither extensive delamination nor burrs of significant size are acceptable in CFRP applications. Both defects often form simultaneously, mainly in the unsupported outer plies of the laminate, and their number increases with tool wear, which makes edge quality control difficult. Burrs can be removed in a separate deburring operation, but this increases manufacturing time and cost [17]. Conventional non-destructive testing methods (ultrasonic, thermographic, radiographic and computed tomography) target internal defects and are applied to the finished component, off-process [9,18,19]. Edge condition is usually assessed visually or microscopically and requires the part to be removed from the machine tool.

Among deburring methods [14], brushing occupies a special place. The flexible brush fibres conform to the edge contour, the machining forces are small and distributed over many fibres, and the process can easily be automated on a machining centre or robot without costly tool positioning systems [20,21,22]. The authors’ previous work addressed deburring and edge shaping of aluminium alloys with ceramic brushes [23], including curvilinear edges machined in multi-axis operations [24], as well as the removal of surface defects [25]. Ceramic-fibre (alumina-based) brushes combine flexibility with abrasive action, and the machining intensity is determined mainly by fibre stiffness and working length [24,26]. The forces in the brush contact zone are a function of fibre deflection and tool engagement depth [27,28], which means that the force signal carries information about the local geometry of the machined edge. Nevertheless, edge brushing of polymer composites has received little attention in the literature.

Process monitoring based on force, vibration or acoustic emission signals enables machining to be assessed in real time [29]. Because signals recorded during the machining of composites are nonlinear and non-stationary (their statistical properties change over time, partly because of material heterogeneity), nonlinear methods, including recurrence plots (RP) and recurrence quantification analysis (RQA), are increasingly used for their analysis [30,31,32]. These methods have been used to detect and locate material defects from the force signal during milling [33,34] and drilling [4]. In GFRP milling, RQA detected defects 0.25 mm in size, approximately 2% of the tool diameter [33], and in drilling, defects 1.2 mm in diameter in GFRP and 1.8 mm in CFRP [4]. Recurrence analysis has also been applied to defect detection in metal forming processes [35]. In parallel, adaptive deburring systems are being developed in which the tool path is generated from a 3D scan of the workpiece [36]; however, these require an additional measurement before machining.

In previous studies, recurrence analysis was used to detect defects hidden in the material during machining with tools of defined cutting-edge geometry [4,33,34]. However, no studies were found in the available literature in which the force signal recorded during brushing was used to detect burrs on the edge. Brushing differs from such processes in that the tool has no defined geometry and the workpiece is contacted simultaneously by many flexible fibres. This study compares the force signal recorded on edge segments with burrs, in the as-trimmed condition, with the signal from chamfered, burr-free segments. It was hypothesised that the two edge conditions differ in the amount of material in the fibre contact zone, which should lead to differences in the character of the force signal that can be revealed by recurrence analysis. If confirmed, this would provide a basis for adaptive deburring, in which the system identifies edge segments requiring additional machining and segments on which the edge has already reached the required condition.

The aim of this study was to assess whether as-milled edges with burrs can be distinguished from burr-free chamfered edges of GFRP and CFRP composites by recurrence analysis of the force signal recorded during brushing, as a first step towards in-process burr detection. Two complementary research tasks were carried out: (i) assessing whether, and from what chamfer size, the burred edge in the as-trimmed condition can be distinguished from the chamfered, burr-free edge on the basis of the force signal, for four ceramic brushes with different fibre stiffness; and (ii) assessing the effect of fibre stiffness on the width of the chamfer produced by brushing. Comparing the two results shows whether the edge condition obtained after brushing falls within the range recognised by the method.

The novelty of this work lies in using the force signal recorded during brushing, a process intended specifically for burr removal, to distinguish the condition of the edges of polymer composites. The results provide a proof of concept for an adaptive deburring system. On burr-free segments, such a system could increase the feed rate, skip an additional brush pass or reduce the brushing depth; on segments with burrs, it would add a tool pass, so that the brushing parameters are adjusted to the actual edge condition.

## 2. Materials and Methods

### 2.1. General Research Methodology

**Figure 1:**
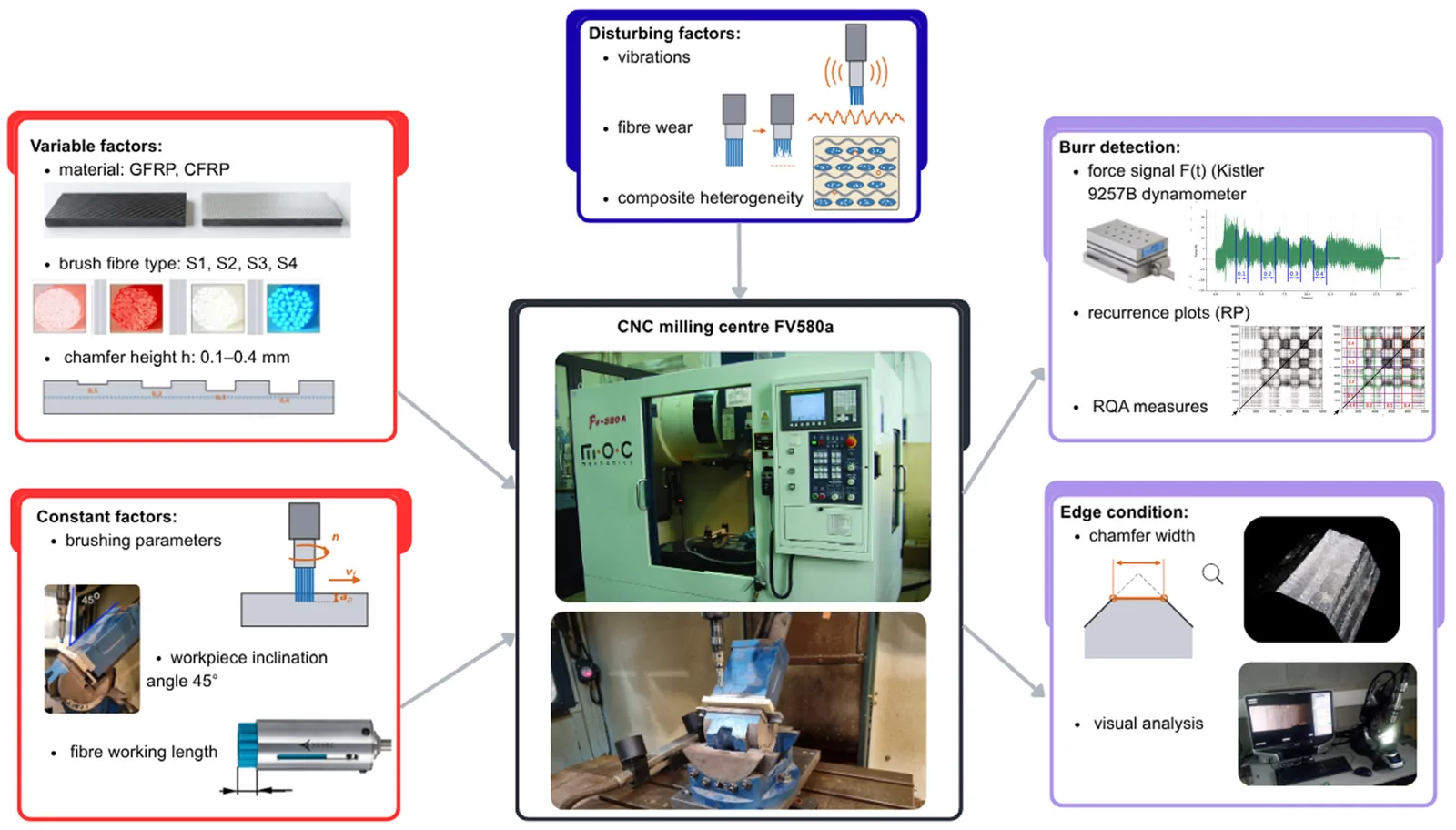
Scheme of the research methodology.

The general research scheme is shown in Figure 1. The edges of GFRP and CFRP specimens were brushed with four ceramic-fibre brushes of increasing stiffness at constant process parameters. Each edge contained alternating segments in the as-milled condition (with burrs) and segments pre-chamfered to a depth of 0.1–0.4 mm, representing a burr-free edge (see Section 2.4.1). Owing to this specimen design, a single tool pass provided two types of data: the force signal, used to distinguish burred segments from chamfered segments, and the edge chamfered by the brush on the burred segments, on which the resulting edge condition was assessed.

### 2.2. Material and Specimens

The tests were carried out on rectangular specimens measuring 10 × 50 × 150 mm, made of epoxy-matrix composites reinforced with glass (GFRP) and carbon (CFRP) fabric. The material characteristics are listed in Table 1. The pre-chamfered segments were produced by milling (see Section 2.4.1).

The prepared specimens were cured in an autoclave for 2.5 h at a temperature of 175 °C and a pressure of 0.4 MPa.

**Figure 2:**
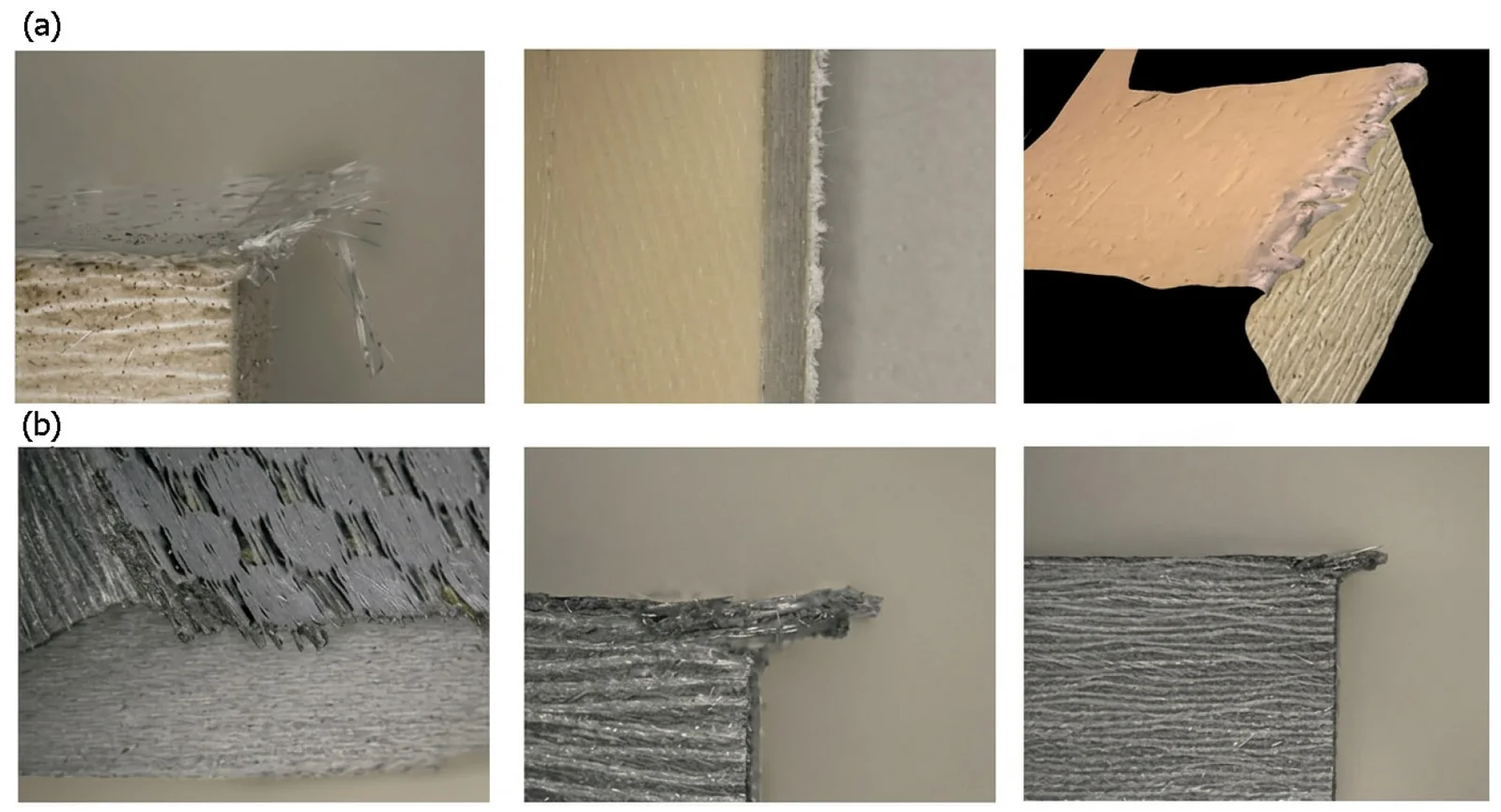
Examples of burrs on the edges of milled fabric-reinforced composites: (**a**) glass (GFRP); (**b**) carbon (CFRP).

After milling, characteristic burrs appeared on the specimen edges; examples are shown in Figure 2.

### 2.3. Tools and Brushing Conditions

Shank-type brushes with ceramic fibres (Xebec Technology Co., Ltd., Tokyo, Japan; types A13 pink, A11 red, A21 white and A32 blue), shown in Table 2, were used. The fibres are held in a sleeve from which they can be extended, changing their working length. Four types of fibres differing in stiffness (S1 < S2 < S3 < S4) were used: pink A13 (S1), intended for machining without changing workpiece dimensions; red A11 (S2), conforming to small shape deviations; white A21 (S3), intended for operation at higher speeds; and blue A32 (S4). The fibre cross-sections were measured with the Keyence VHX 5000 microscope (Keyence Corporation, Osaka, Japan); as the cross-sections are oval, both the major and the minor axis were measured (Table 2).

**Figure 3:**
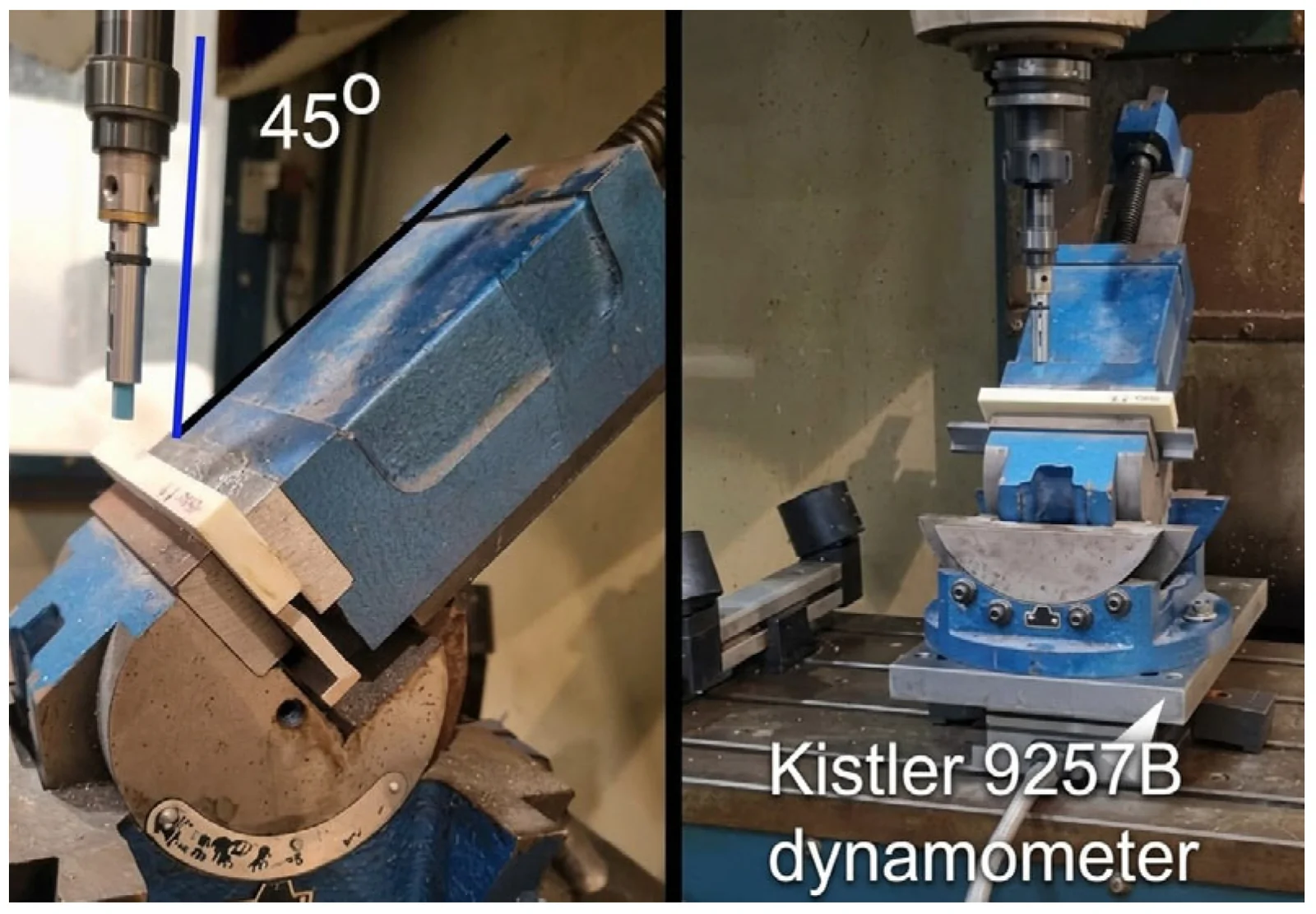
Position of the workpiece relative to the tool (45° angle) and clamping on the Kistler 9257B dynamometer.

Brushing was carried out dry on an FV580a vertical machining centre (MOC Mechanicy, Pruszków, Poland) at the constant parameters listed in Table 3, which were selected on the basis of preliminary tests and the recommendations of the brush manufacturer. The specimen was clamped in a tilting vice mounted on the dynamometer so that both surfaces forming the edge were inclined at 45° to the tool axis (Figure 3). The brushing depth was set at a constant a_p_ = 0.5 mm, measured from the nominal specimen edge along the tool axis. Before each test, the cutting ability of the brush was restored by grinding the fibre end face, and the fibre extension from the sleeve was readjusted to 10 mm.

### 2.4. Edge-Condition Discrimination Methodology

#### 2.4.1. Segments with Burrs and Chamfered Segments

**Figure 4:**
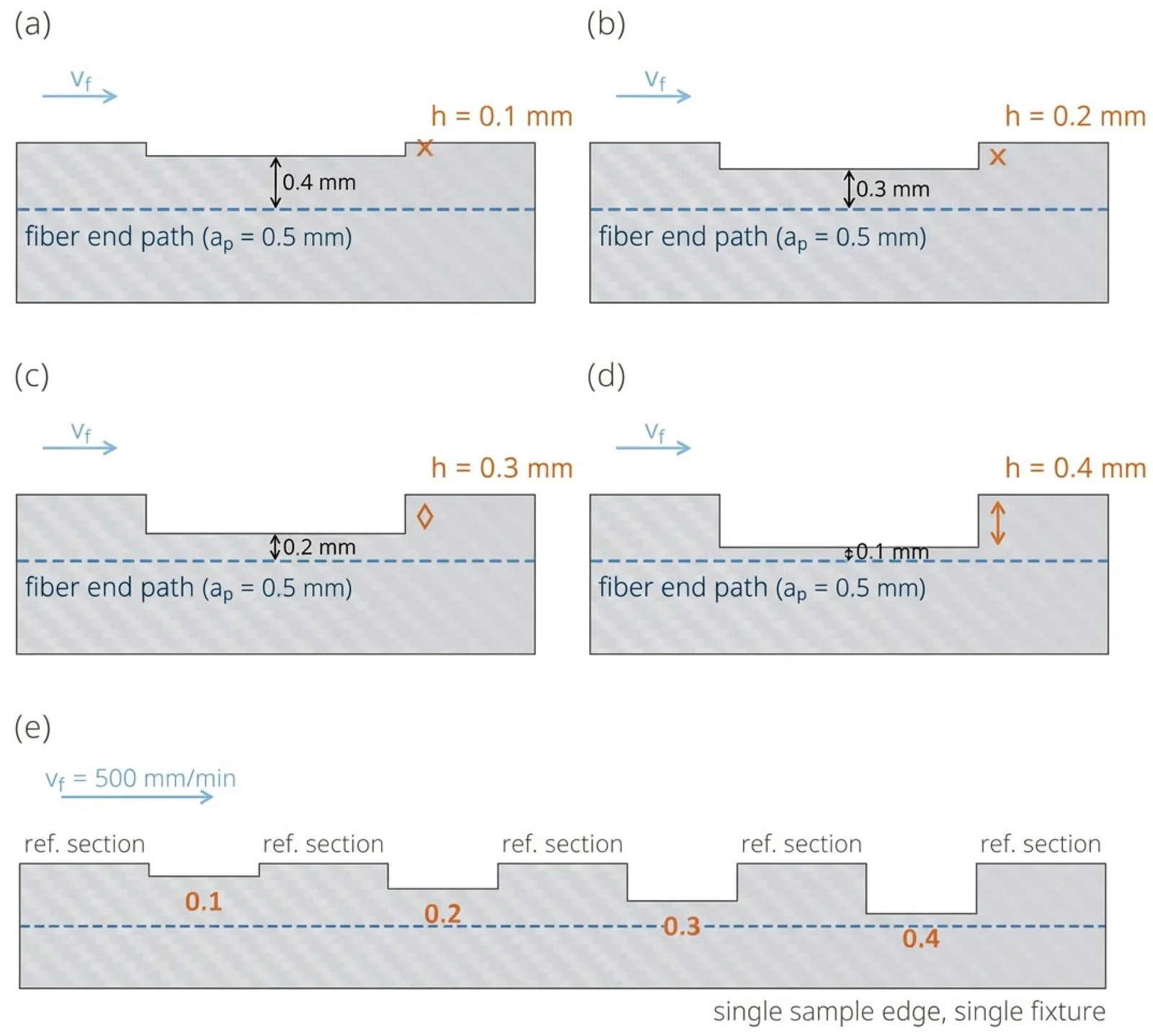
Chamfered segments on the specimen edge: (**a**–**d**) sections along the edge for *h* = 0.1, 0.2, 0.3 and 0.4 mm, with the dashed line indicating the path of the fibre tips (*a_p_* = 0.5 mm); (**e**) arrangement of chamfered segments and burred segments on one specimen edge (vertical scale exaggerated).

Burrs formed during the edge trimming of composites are random in shape, size and distribution (Figure 2); therefore, they cannot be produced reproducibly. On the as-milled edges of the tested specimens, burrs occurred irregularly along the edge, and their height ranged from approx. 0.1 mm to approx. 2.0 mm. For this reason, two edge conditions were compared. Edge segments in the as-trimmed condition, sharp and with burrs, were treated collectively as the burred edge. Segments pre-chamfered with a 12 mm diameter end mill to a depth of *h* = 0.1, 0.2, 0.3 and 0.4 mm, measured along the tool axis, were taken as the burr-free edge (Figure 4). With the surfaces forming the edge inclined at 45°, the chamfer width is approximately twice its depth *h* (0.2–0.8 mm), which corresponds to the edge condition obtained by brushing (Section 3.2). As in defect detection studies using defects of predefined geometry [4,33], this enabled a controlled assessment of the sensitivity of the method.

**Figure 5:**
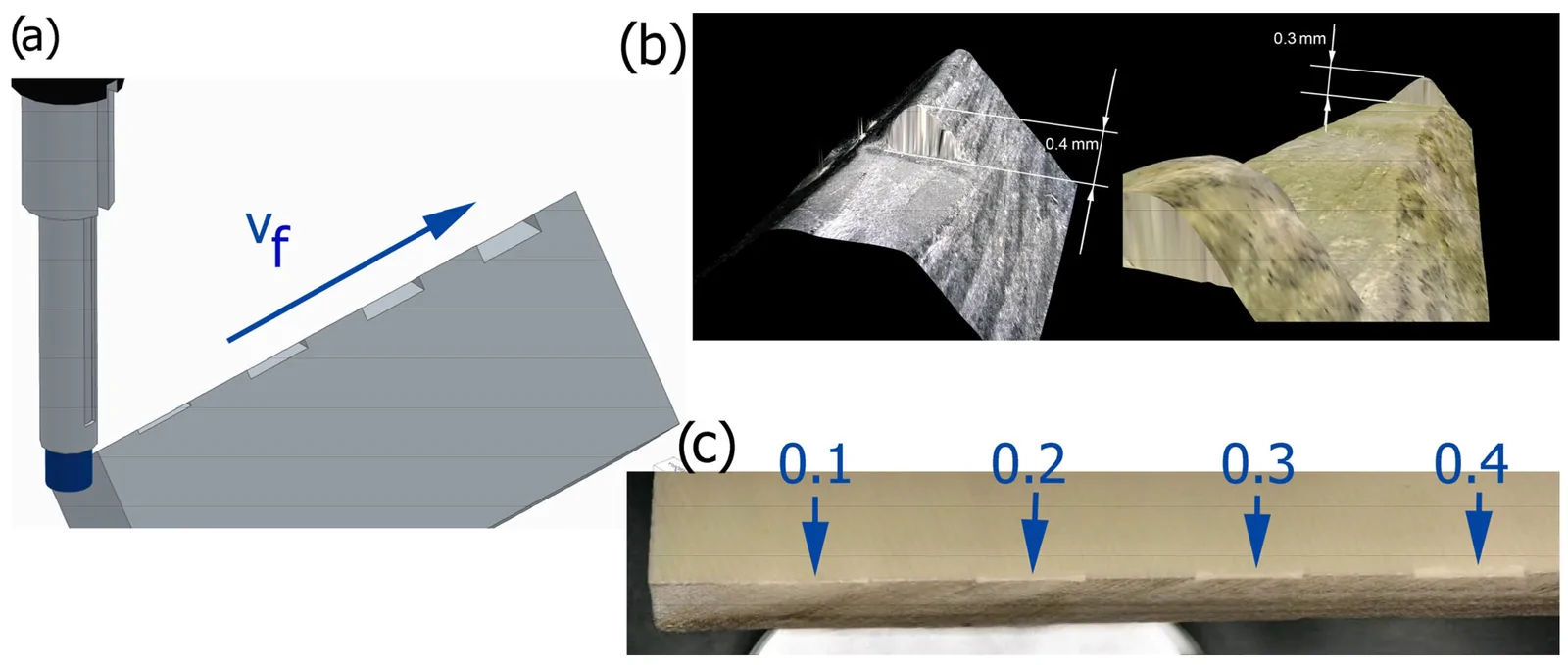
Chamfered segments on the edge: (**a**) 3D model with the feed direction indicated; (**b**) 3D image of chamfers 0.4 mm and 0.3 mm deep (Keyence VHX 5000 microscope); (**c**) actual view of a GFRP specimen with the chamfers.

The geometry of the chamfered segments is shown in Figure 4a–d. The fibre tips move along a path offset by a_p_ = 0.5 mm from the nominal edge. On a chamfered segment of depth h, the fibre engagement in the material decreases to *a_p_ − h* (0.4, 0.3, 0.2 and 0.1 mm, respectively), and the amount of material removed is smaller than on the burred segments. The model and the actual view of the chamfered segments are shown in Figure 5.

All chamfered segments were made on one edge of each specimen, and the force signal for all chamfer depths was recorded in a single tool pass and a single clamping. There were two reasons for this. First, the smallest chamfer depth tested (0.1 mm) is 20% of a_p_, so an error in setting a_p_ after re-clamping could significantly affect the measurement results. Second, making the chamfers on four separate specimens would introduce differences in clamping conditions, which, as shown for composite drilling, affect machining accuracy and defect formation [37]. With a single edge, all segments are machined with identical clamping, the same a_p_ reference and the same fibre condition, while the burred segments between them provide a local signal reference level. A similar approach (multiple defects in one clamping) was used in previous defect detection studies [4,33]. For each material–brush combination, three specimens were brushed in a single pass; each edge therefore contained four chamfered segments and five as-milled segments.

#### 2.4.2. Force Measurement

Forces were measured on the machining centre with a three-component piezoelectric dynamometer (type 9257B, Kistler Instrumente AG, Winterthur, Switzerland) connected to a Kistler type 5070 charge amplifier. Signals were recorded using a DynoWare (type 2825A) data acquisition system (type 5697A) and DynoWare software (type 2825A). The main element of the system is the 3D dynamometer, which measures the brushing forces. The second element is the charge amplifier, which converts the electric charge into a proportional voltage. The data acquisition system connects and controls the charge amplifiers and works with a PC running the general-purpose DynoWare software. The recorded signal covered the entire tool pass along the edge (approx. 20 s) and was sampled at 10 kHz. The section covering the contact of the brush with the material was selected for analysis. The analysis used the Fz force component, acting along the tool axis. Recurrence analysis was performed using the cutting force signals without additional digital filtering or amplitude normalisation. This approach was adopted to preserve the original time-domain signals recorded during the machining process. Since a constant recurrence rate (RR = 0.20) was applied, the distance threshold ε was individually adjusted for each analysed signal segment. Avoiding additional digital filtering made it possible to prevent modifications to the frequency components of the signal, which may contain information about the dynamics of the cutting process.

#### 2.4.3. Recurrence Analysis

Since a single quantity was measured, the phase space was reconstructed using the time-delay method. For a series x_1_, x_2_, …, x_N_, the following vectors are constructed:

X_i_ = (x_i_, x_i+τ_, x_i+2τ_, …, x_i+(m−1)τ_), i = 1, 2, …, n, (1)

where τ is the time delay and m is the embedding dimension, usually determined by the average mutual information (AMI) and false nearest neighbours (FNN) methods, respectively [38,39]. The phase space reconstruction parameters were selected using the Average Mutual Information (AMI) method and the False Nearest Neighbours (FNN) method. These methods are used to determine the time delay and embedding dimension, respectively, which are required to reconstruct the phase space from a one-dimensional time series. In the AMI method, the average mutual information is calculated for successive time delays, and the resulting function is then analysed. The time delay used for phase space reconstruction is generally selected as the value corresponding to the first distinct local minimum of the AMI function. Once the time delay has been determined, the embedding dimension is estimated using the FNN method. This method involves analysing changes in the distances between nearest neighbours in phase spaces with successively increasing dimensions. False nearest neighbours are defined as points whose apparent proximity results from representing the system dynamics in a space with an insufficient dimension. The appropriate embedding dimension is selected as the value at which the proportion of false nearest neighbours reaches a sufficiently low level or stabilises. The recurrence plot is a symmetric binary matrix:

R_i,j_(ε) = Θ(ε − ‖X_i_ − X_j_‖), i, j = 1, 2, …, n, (2)

where Θ is the Heaviside function, ε is the threshold parameter and ‖·‖ is the Euclidean norm [30,31]. The choice of the threshold ε affects the RP structure and the values of the measures, especially in the presence of measurement noise [40,41]. In this study, m = 6 and τ = 5 were adopted. The calculations were performed for a fixed recurrence rate RR = 0.2, so the threshold ε was adjusted individually for each analysed fragment to give 20% recurrence points. Fixing RR removes the effect of the overall signal amplitude on the density of recurrence points; the RQA measures based on line structures (determinism, DET; average diagonal line length, L; laminarity, LAM; trapping time, TT) and the recurrence time of the second type (T2) therefore describe the arrangement of recurrence points rather than the signal level, and differences in RP density between separately thresholded fragments are not interpreted. The recurrence quantification analysis (RQA) was performed using the CRP Toolbox for MATLAB R2025b. The minimum length of diagonal and vertical lines was set to 2, while the Theiler window was set to 1. The analysis was carried out using a window size of 500 samples and a window step of 1 sample. In the RPs, the force signal corresponds to the main diagonal (direction indicated by an arrow), and the intervals corresponding to the location of the chamfered segments were marked on the basis of the brush transit time.

RQA measures (Table 4) [31,32,42] were used to quantify the RP structures. They were computed separately for the signal fragments corresponding to each chamfered segment and for reference fragments of the same length recorded on the burred edge, so that the values of the measures could be related to the edge condition. The reference fragments were taken from stable portions of the signal on the as-milled segments of the same pass; the brush entry and exit and the transition zones adjacent to the chamfer boundaries were excluded.

### 2.5. Edge Condition Assessment

**Figure 6:**
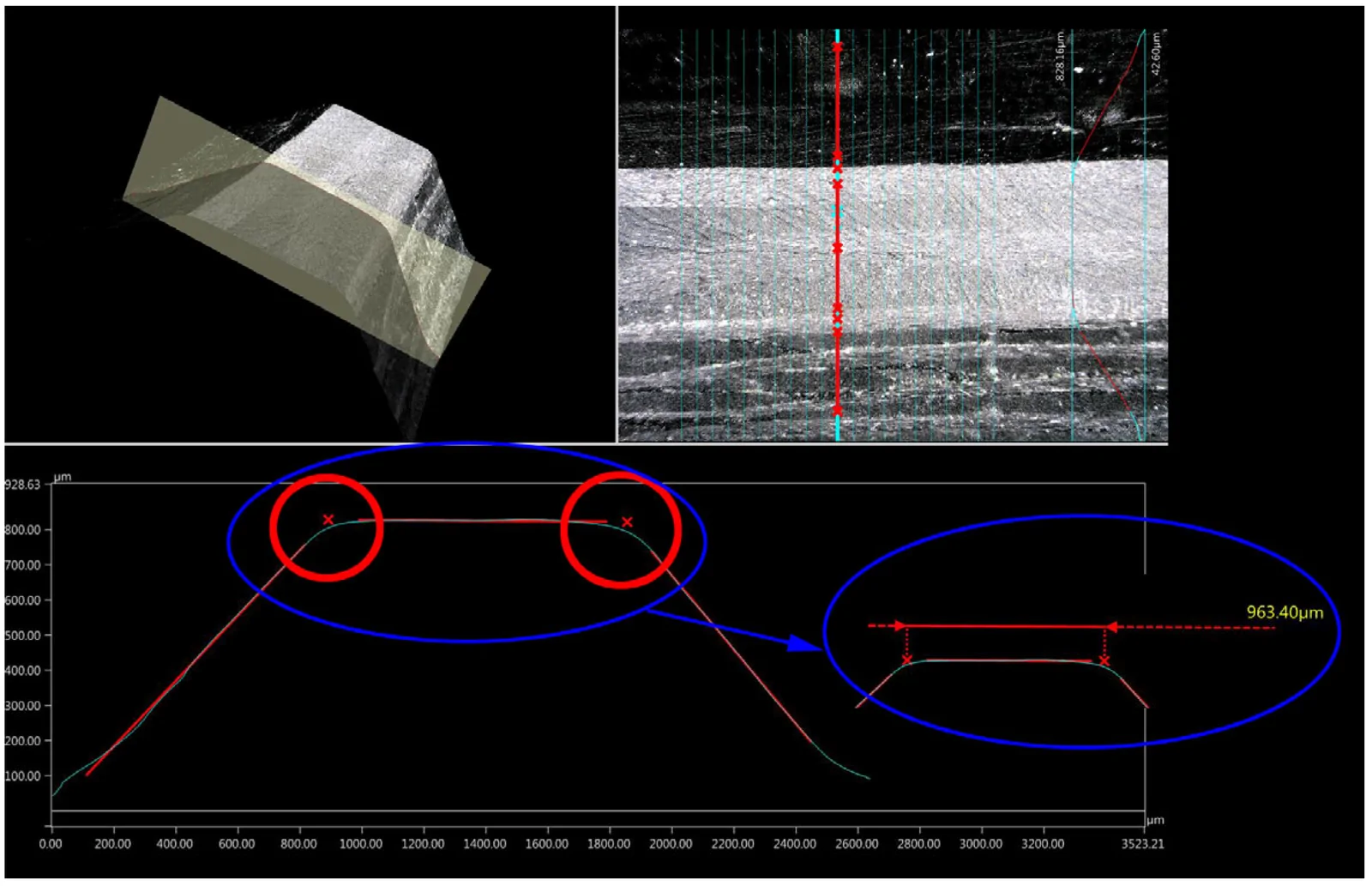
Method of determining the chamfer width after brushing: 3D model of the edge, selection of the section and measurement on the profile.

The edge condition after brushing was assessed on the segments that were in the as-milled condition before brushing (without a pre-chamfer) using a Keyence VHX 5000 digital microscope (Keyence Corporation, Osaka, Japan). Using the function of stacking images taken at different focal planes, a 3D model of the edge was created, followed by a profile in the section perpendicular to the edge (Figure 6). The chamfer width b_f_ was determined as the distance between the points where the chamfer profile meets the surfaces forming the edge. For each material–brush combination, three specimens were brushed in a single pass and five measurements were taken on each specimen at ×300 magnification at locations distributed along the whole length of the edge, on the as-milled segments between the chamfered segments.

## 3. Results

### 3.1. Discrimination of Edge Conditions

#### 3.1.1. Force Signal

**Figure 7:**
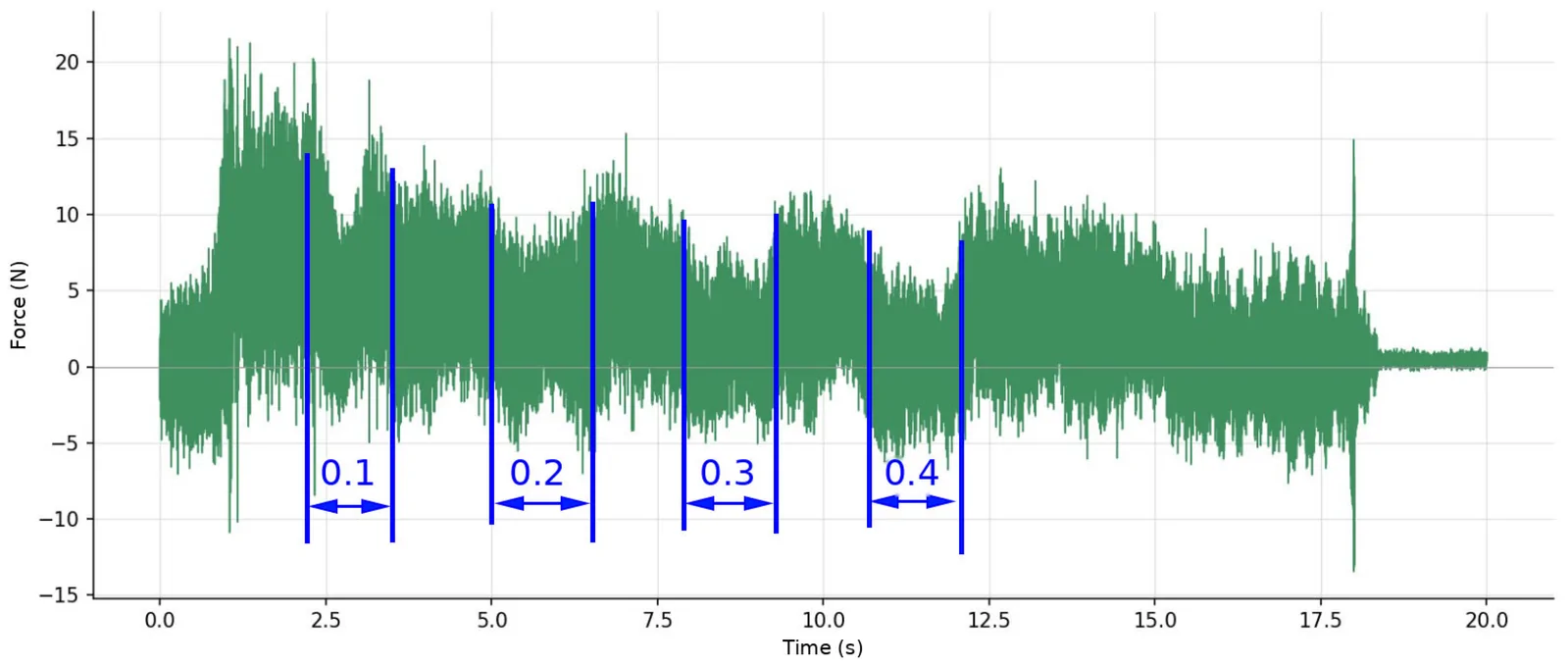
Example of the force signal during a pass of brush S4 along a CFRP edge with chamfered segments 0.1–0.4 mm deep.

An example of the force signal recorded during brushing of a CFRP edge with brush S4 is shown in Figure 7. Pulses corresponding to the brush entering and leaving the material are visible at the beginning and end of the signal. On the chamfered segments, the force amplitude is lower than on the burred segments, owing to the reduction in fibre engagement to *a_p_−h*. These differences are clear for chamfers 0.2–0.4 mm deep. For 0.1 mm, they are comparable to the natural variability of the signal resulting from the stochastic contact of many fibres with the heterogeneous material.

#### 3.1.2. Recurrence Plots for GFRP

**Figure 8:**
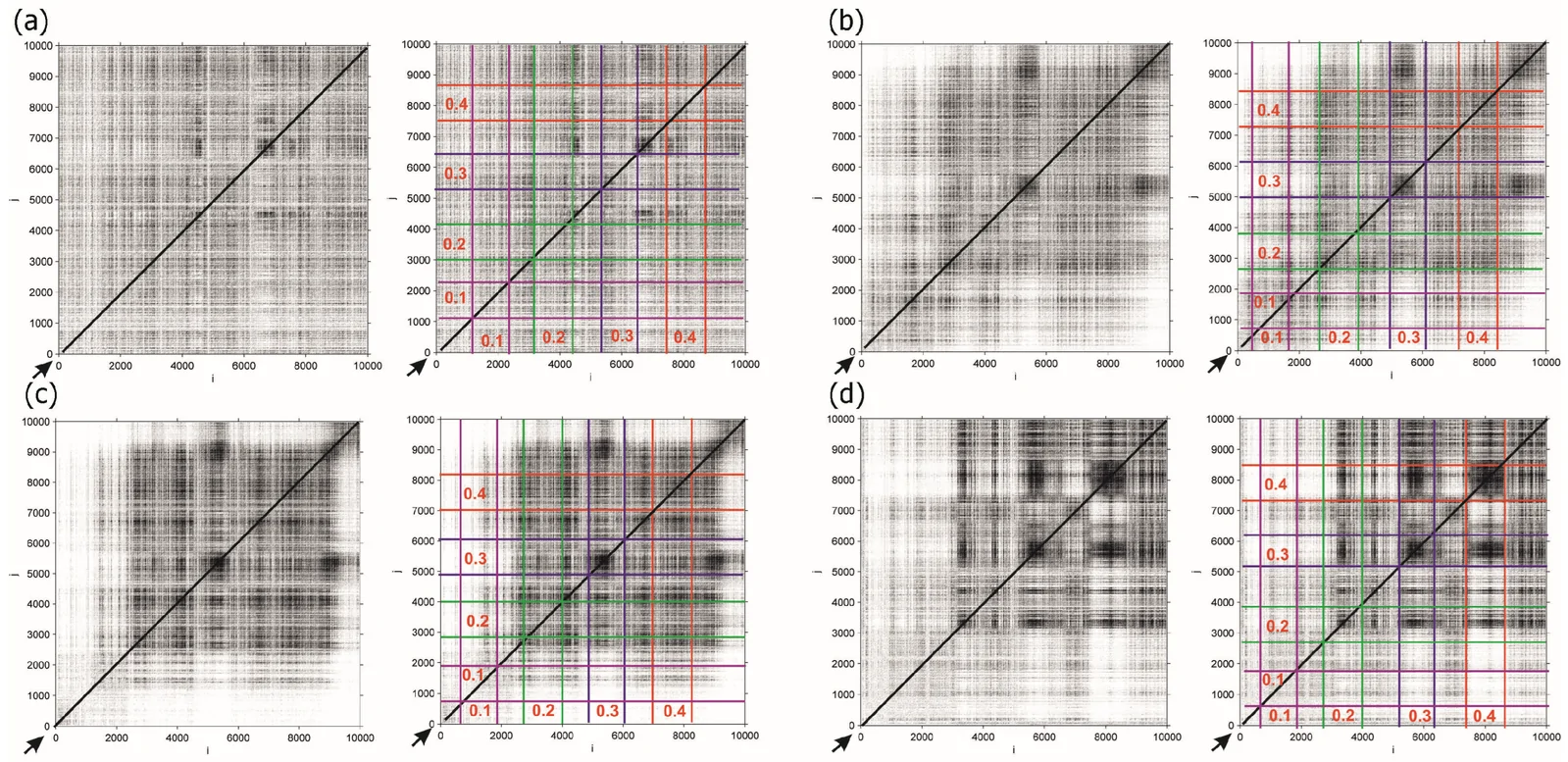
Recurrence plots of the force signal during brushing of a GFRP edge with brushes: (**a**) S1; (**b**) S2; (**c**) S3; (**d**) S4. In each pair, the left plot shows the RP and the right plot shows the intervals of chamfers 0.1–0.4 mm deep. Symbols “i” and “j” correspond to the indices of successive states of the dynamic system under analysis, determined based on the measurement signal.

The recurrence plots of the force signal for GFRP are shown in Figure 8 (left: original plot; right: with the chamfered-segment intervals marked). For brush S1 (lowest stiffness), vertical and horizontal bands and areas of varying recurrence point density appear throughout the plot, but they do not correspond to the location of the 0.1–0.4 mm chamfered segments, and the intensity of the changes does not increase with h. For brush S2, the plot is segmented into areas of different density; the intervals of the 0.3 and 0.4 mm chamfered segments coincide with areas of increased density and altered recurrence structure. For brush S3, the plot has a distinct block structure: the density of recurrence points increases in the interval of the 0.2 mm chamfer, and the most characteristic response was obtained for 0.3 mm. For brush S4 (highest stiffness), a change in the character of recurrence is visible for 0.2, 0.3 and 0.4 mm. The 0.4 mm chamfer produces a compact, high-density structure near the main diagonal. For none of the brushes was the 0.1 mm chamfered segment unambiguously recognisable.

#### 3.1.3. Recurrence Plots for CFRP

**Figure 9:**
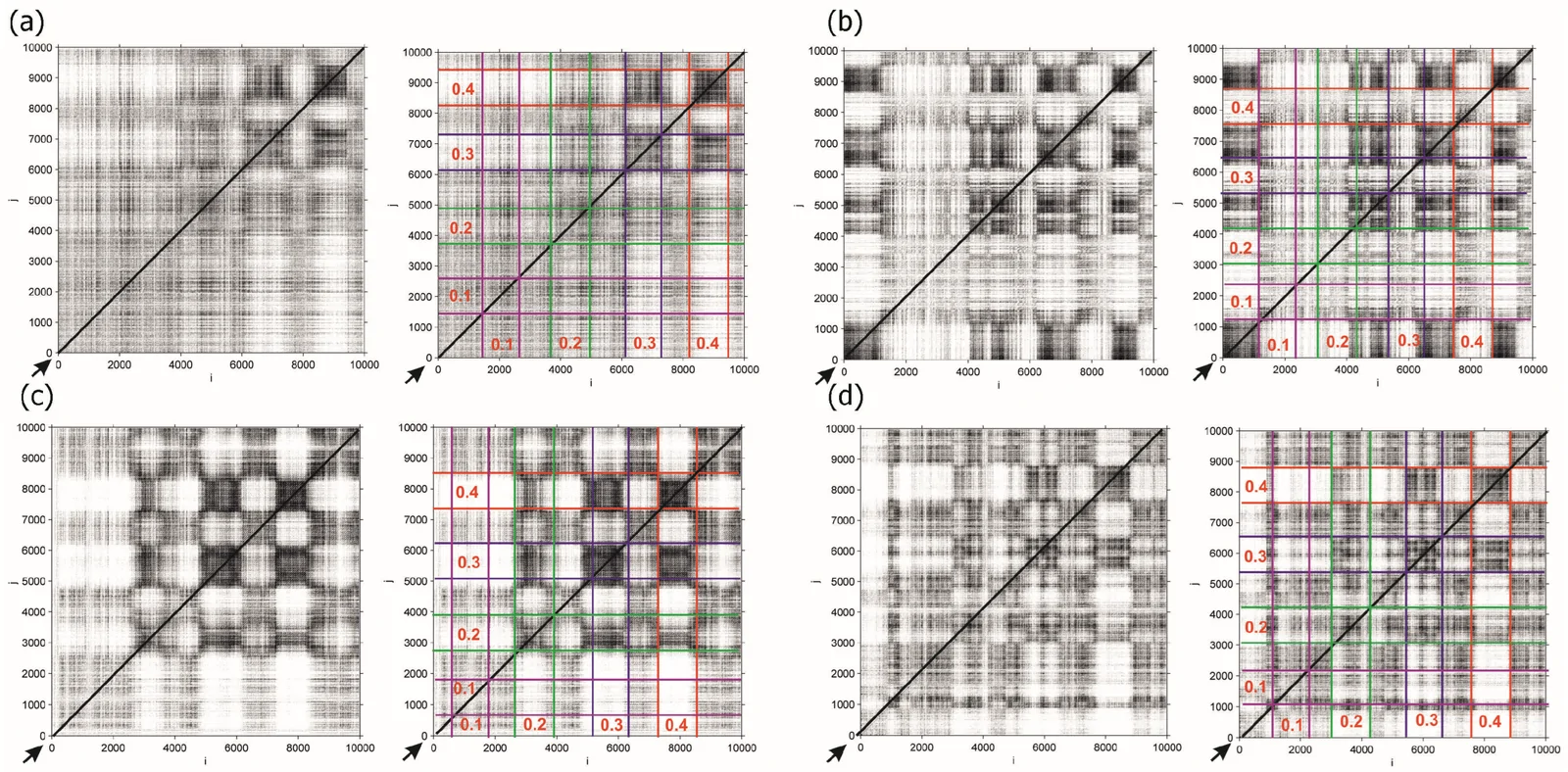
Recurrence plots of the force signal during brushing of a CFRP edge with brushes: (**a**) S1; (**b**) S2; (**c**) S3; (**d**) S4. In each pair, the left plot shows the RP and the right plot shows the intervals of chamfers 0.1–0.4 mm deep. Symbols “i” and “j” correspond to the indices of successive states of the dynamic system under analysis, determined based on the measurement signal.

For CFRP (Figure 9), local changes in recurrence density occur in the intervals of the 0.2 and 0.3 mm chamfered segments already with brush S1, and the best agreement between the chamfer location and the RP densification was obtained for 0.4 mm. Brush S2 produces a distinct block structure with higher contrast than S1; the changes for 0.2 mm do not clearly correspond to the chamfer boundaries, while those for 0.3 and 0.4 mm are distinct. For brush S3, the structural change in the 0.2 mm interval corresponds better to the chamfer boundaries than for the less stiff brushes, and chamfers 0.3 and 0.4 mm deep form compact, high-density areas near the main diagonal. For brush S4, a slight change was observed even for 0.1 mm, increased recurrence activity for 0.2 mm and compact clusters of points for 0.3 and 0.4 mm, with the most characteristic response for 0.4 mm.

The visual assessment of the recognisability of the chamfered segments in the RPs is summarised in Table 5. Distinguishability increases with chamfer depth and fibre stiffness, and the contrast of the structures is generally greater for CFRP than for GFRP.

#### 3.1.4. Recurrence Quantification Analysis

Recurrence plots provide only qualitative information. Therefore, recurrence quantification analysis (RQA) was used to quantitatively distinguish burred segments from chamfered segments. Of the recurrence measures analysed, those most sensitive to changes in chamfer depth were selected and are presented here: determinism (DET), average diagonal line length (L), laminarity (LAM), trapping time (TT) and the recurrence time of the second type (T2). Their values were determined for the two composite materials (GFRP and CFRP), the four brushes (S1–S4) and chamfer depths *h* of 0.1–0.4 mm. The results were compared with the reference values for the burred edge, which are marked with dashed lines in the plots.

**Figure 10:**
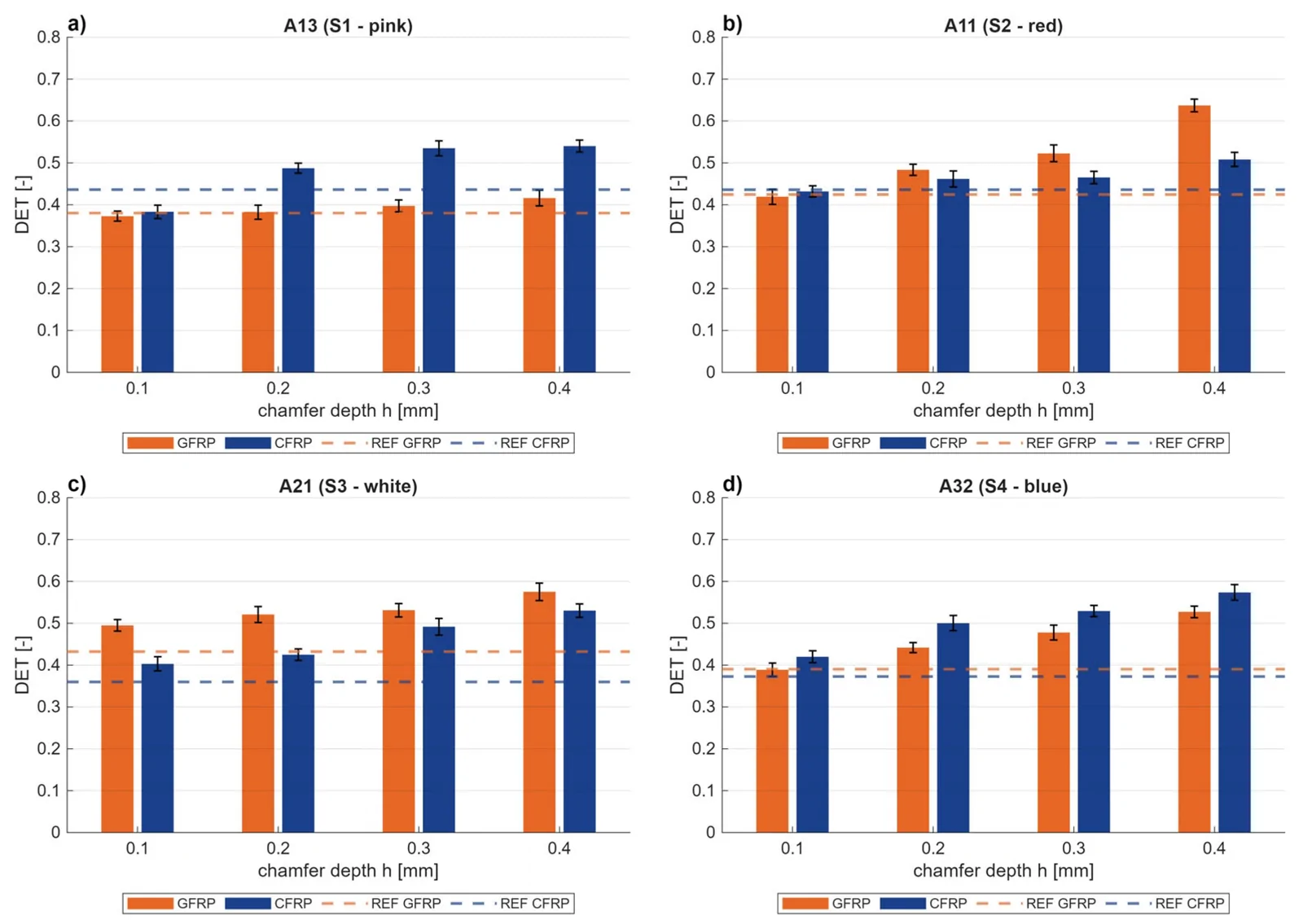
DET as a function of chamfer depth *h* for the two materials and brushes: (**a**) S1 (A13); (**b**) S2 (A11); (**c**) S3 (A21); (**d**) S4 (A32).

Figure 10 shows the changes in determinism (DET), defined as the fraction of recurrence points forming diagonal lines in the total number of recurrence points.

This measure characterises the degree to which repetitive, deterministic structures occur in the analysed signal. For most brushes, DET increased with chamfer depth, and whether the reference level was exceeded depended on the brush and the material. For brush S1 (Figure 10a), DET increased slightly for GFRP and exceeded the reference level from a chamfer depth of 0.3 mm. For CFRP, the value at 0.1 mm remained below the reference level, whereas from 0.2 mm it clearly exceeded it. For brush S2 (Figure 10b), DET increased for GFRP, particularly at a chamfer depth of 0.4 mm, whereas for CFRP the changes were smaller; for both materials, the reference levels were exceeded from 0.2 mm. A similar increasing trend for GFRP was found for brush S3 (Figure 10c), for which the DET values remained above the reference level for all chamfer depths. For CFRP, the reference level was also exceeded over the whole range, although the increase in the measure was less pronounced. For brush S4 (Figure 10d), DET increased with chamfer depth for both materials, more markedly for CFRP. For CFRP, the reference level was exceeded over the whole range of chamfer depths, and for GFRP from 0.2 mm. These results indicate that DET makes it possible to identify changes related to chamfer depth; the smallest chamfers were best detected with brush S3 for both materials and with brush S4 for CFRP.

**Figure 11:**
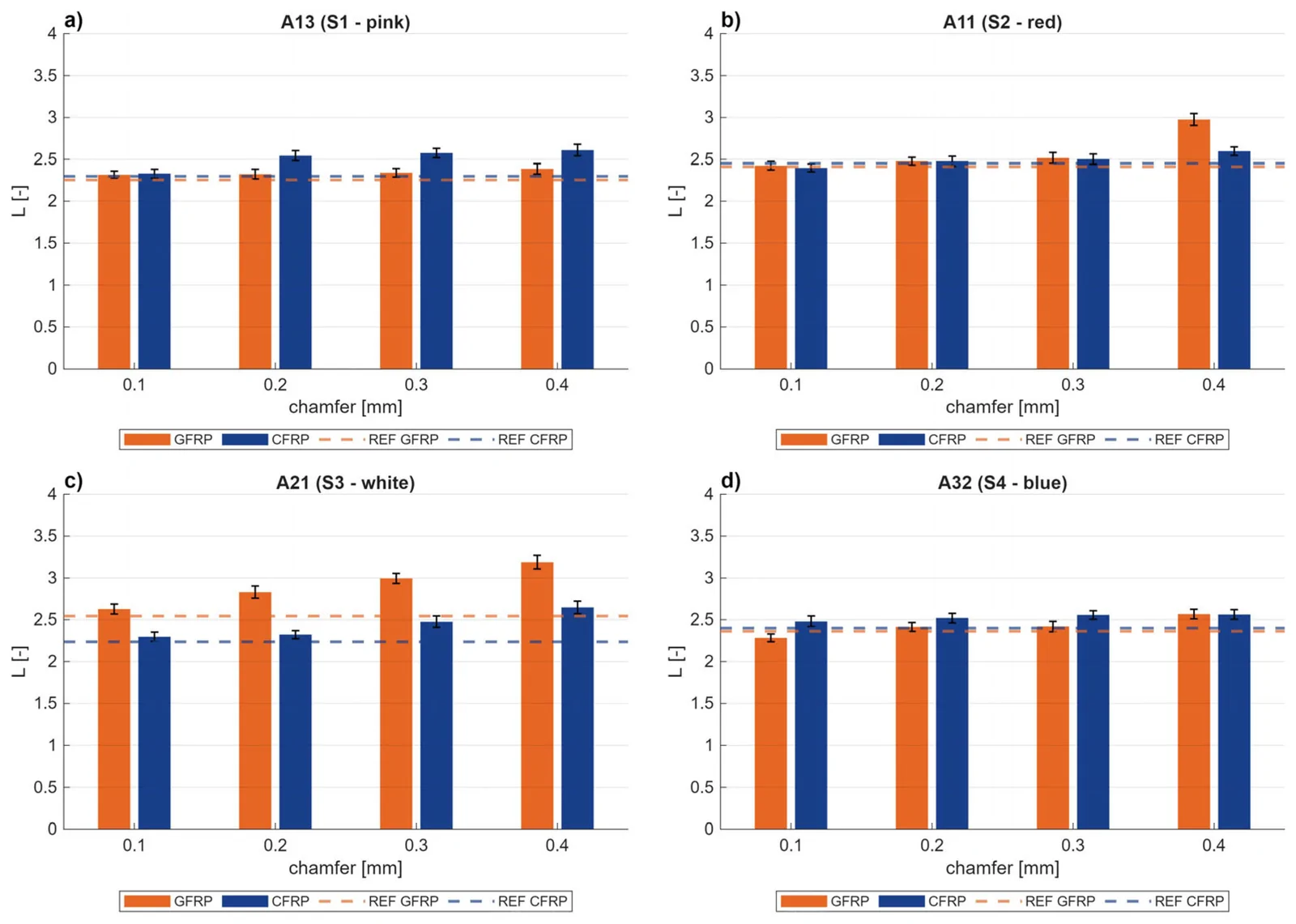
L as a function of chamfer depth *h* for the two materials and brushes: (**a**) S1 (A13); (**b**) S2 (A11); (**c**) S3 (A21); (**d**) S4 (A32).

Figure 11 shows the values of L, the average length of diagonal lines in the recurrence plot.

This parameter describes the average length of the intervals in which the analysed system shows a similar evolution of states. In most cases, L increased with chamfer depth, and the extent to which the reference levels were exceeded depended on the brush and the material. For brush S1 (Figure 11a), the values of L for GFRP changed only slightly and remained only marginally above the reference level. For CFRP, the value at 0.1 mm was close to the reference level, whereas from 0.2 mm the reference level was clearly exceeded. For brush S2 (Figure 11b), L increased for GFRP, particularly at a chamfer depth of 0.4 mm, where it reached approx. 3.0, clearly exceeding the reference level. For CFRP, the changes in L were smaller, and the reference level was exceeded from 0.2 mm. The largest changes in L for GFRP occurred for brush S3 (Figure 11c), for which the measure increased from approx. 2.63 to 3.18, and all values exceeded the reference level. For CFRP, the values of L were also above the reference level over the whole range and increased with chamfer depth. For brush S4 (Figure 11d), L increased for both materials; for CFRP it remained above the reference level over the whole range, whereas for GFRP the reference level was exceeded from 0.2 mm. These results indicate that L makes it possible to identify changes related to chamfer depth; the highest sensitivity was found for GFRP with brush S3, whereas for CFRP the reference levels were most clearly exceeded with brushes S1, S3 and S4.

**Figure 12:**
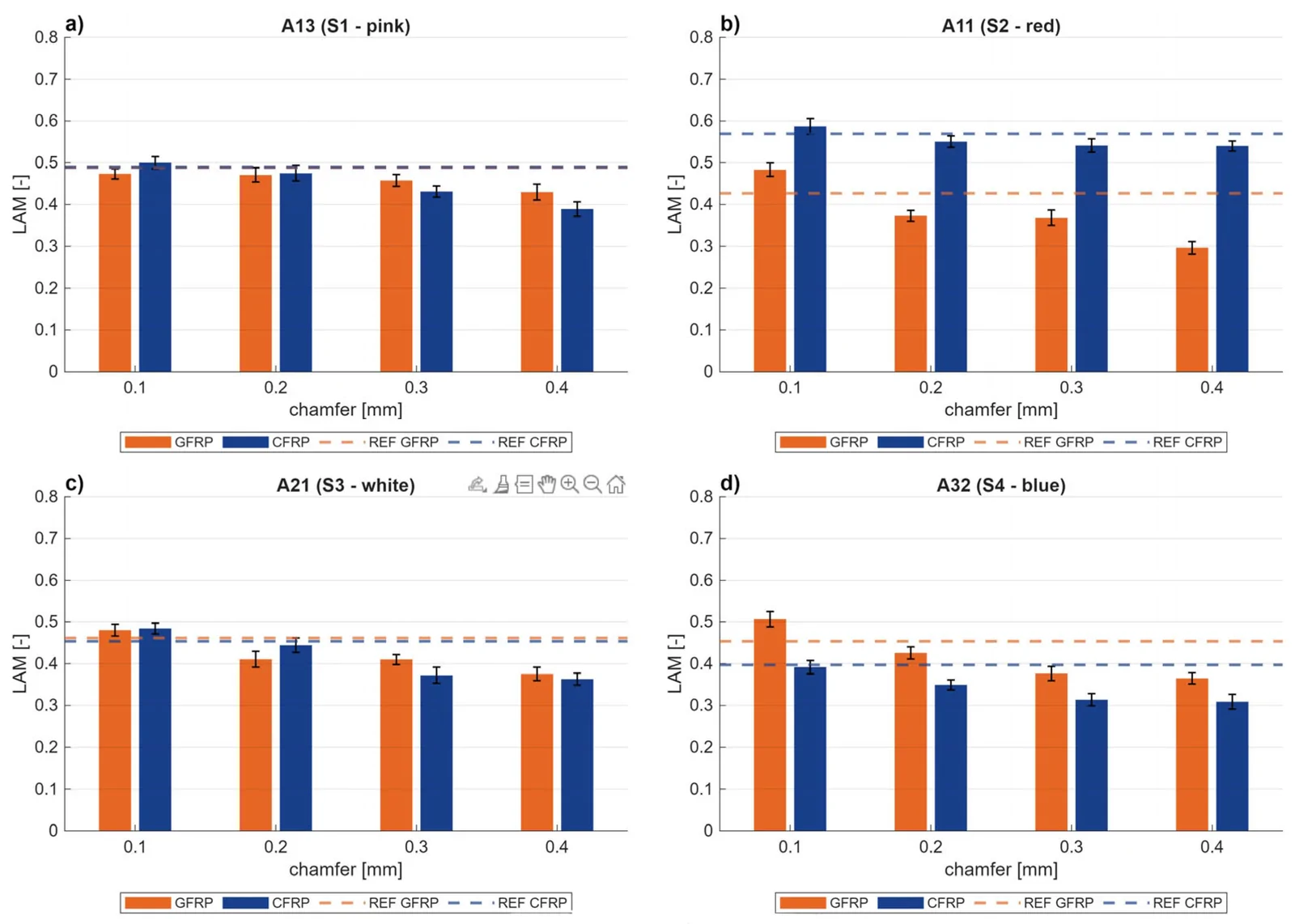
LAM as a function of chamfer depth *h* for the two materials and brushes: (**a**) S1 (A13); (**b**) S2 (A11); (**c**) S3 (A21); (**d**) S4 (A32).

Figure 12 shows the changes in laminarity (LAM), the fraction of recurrence points forming vertical lines.

This measure describes the occurrence of states in which the dynamics of the analysed system remain similar for some time. In contrast to DET and L, LAM mostly decreased with increasing chamfer depth, and in most cases its values remained below the reference levels. For brush S1 (Figure 12a), the LAM values for GFRP showed a slight decreasing trend and remained below the reference level over the whole range. For CFRP, the value at 0.1 mm slightly exceeded the reference level, whereas from 0.2 mm it remained below it, with the deviation increasing with chamfer depth. For brush S2 (Figure 12b), a clear decrease in LAM was observed for GFRP, with values below the reference level from 0.2 mm. For CFRP, LAM also decreased, and the reference level was exceeded only at 0.1 mm. For brush S3 (Figure 12c), LAM decreased with increasing chamfer depth for both materials: at 0.1 mm the values for GFRP and CFRP exceeded the respective reference levels, whereas from 0.2 mm they remained below them. For brush S4 (Figure 12d), a clear decreasing trend was observed for both materials. For GFRP, LAM at 0.1 mm exceeded the reference level, whereas from 0.2 mm it remained below it; for CFRP, the values were below the reference level over the whole range. These results indicate that LAM makes it possible to identify changes related to chamfering, with the largest deviations from the reference levels observed for the larger chamfer depths. Particularly pronounced changes occurred for GFRP with brushes S2 and S4 and for CFRP with brush S4.

**Figure 13:**
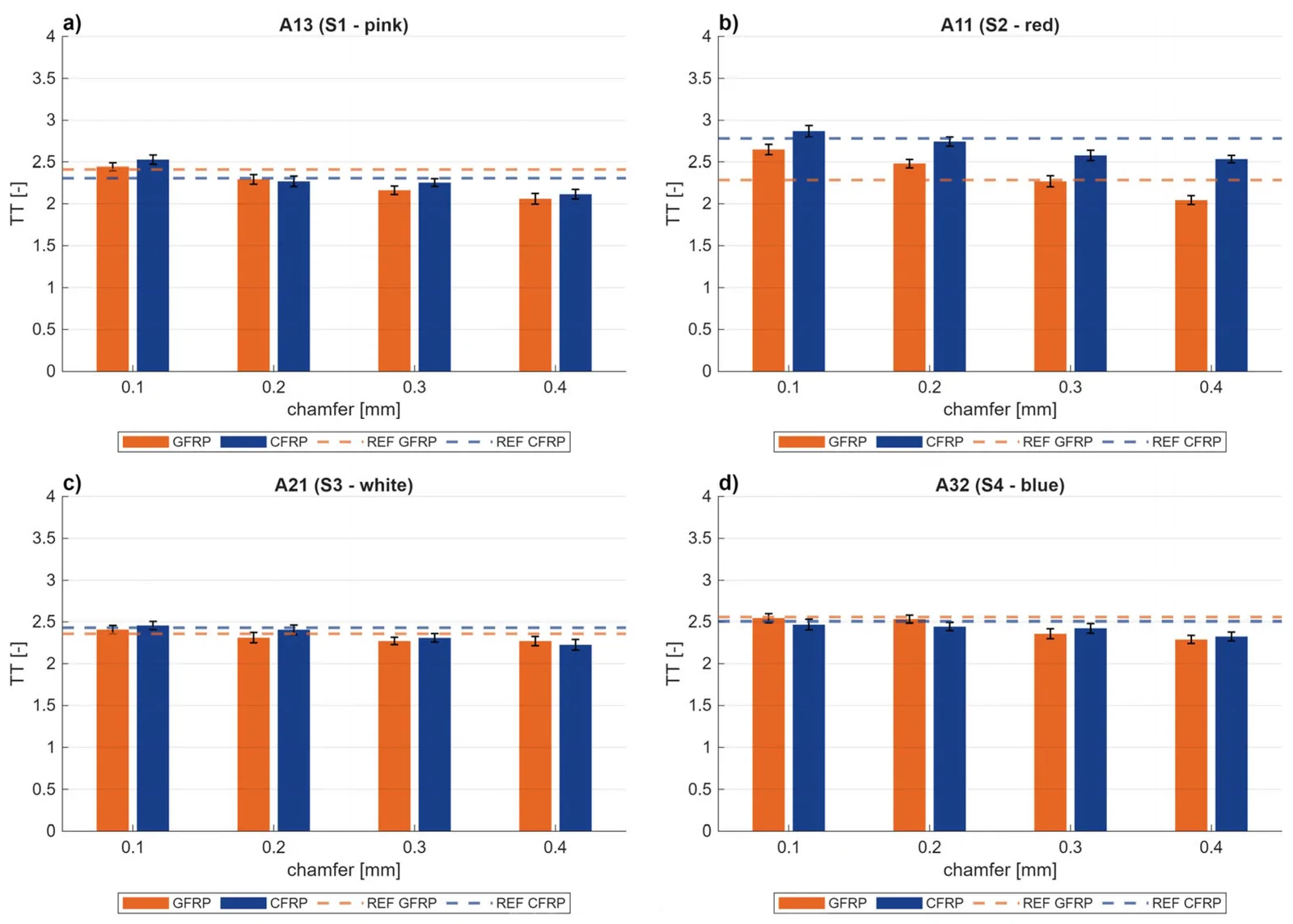
TT as a function of chamfer depth *h* for the two materials and brushes: (**a**) S1 (A13); (**b**) S2 (A11); (**c**) S3 (A21); (**d**) S4 (A32).

Figure 13 shows the values of the trapping time (TT), the average length of vertical lines in the recurrence plot.

This parameter characterises the average time for which the system remains in states with small changes in dynamics. In most cases, TT decreased with increasing chamfer depth, and the magnitude of the deviations from the reference levels depended on the brush and the material. For brush S1 (Figure 13a), TT gradually decreased for both materials. For GFRP, the value at 0.1 mm slightly exceeded the reference level, whereas from 0.2 mm it remained below it. For CFRP, the reference level was also exceeded at 0.1 mm, while for greater depths the values of TT remained below it. A similar decreasing trend was observed for brush S2 (Figure 13b), more pronounced for GFRP than for CFRP. For GFRP, TT exceeded the reference level at 0.1 and 0.2 mm and remained below it from 0.3 mm; for CFRP, the values remained below the reference level from 0.2 mm. For brush S3 (Figure 13c), TT changed only slightly, with a small decreasing tendency for both materials: at 0.1 mm the values for GFRP and CFRP were close to the respective reference levels, whereas for greater depths they remained below them. For brush S4 (Figure 13d), TT gradually decreased for both materials. For GFRP, the values at 0.1 and 0.2 mm were close to the reference level, whereas from 0.3 mm a clearer deviation below it occurred; for CFRP, TT remained below the reference level over the whole range, and its decrease with chamfer depth was less pronounced. These results indicate that TT makes it possible to identify changes related to chamfering, particularly at larger chamfer depths. The largest deviations from the reference levels were observed for GFRP with brushes S1 and S2, and for CFRP with brushes S1 and S2.

**Figure 14:**
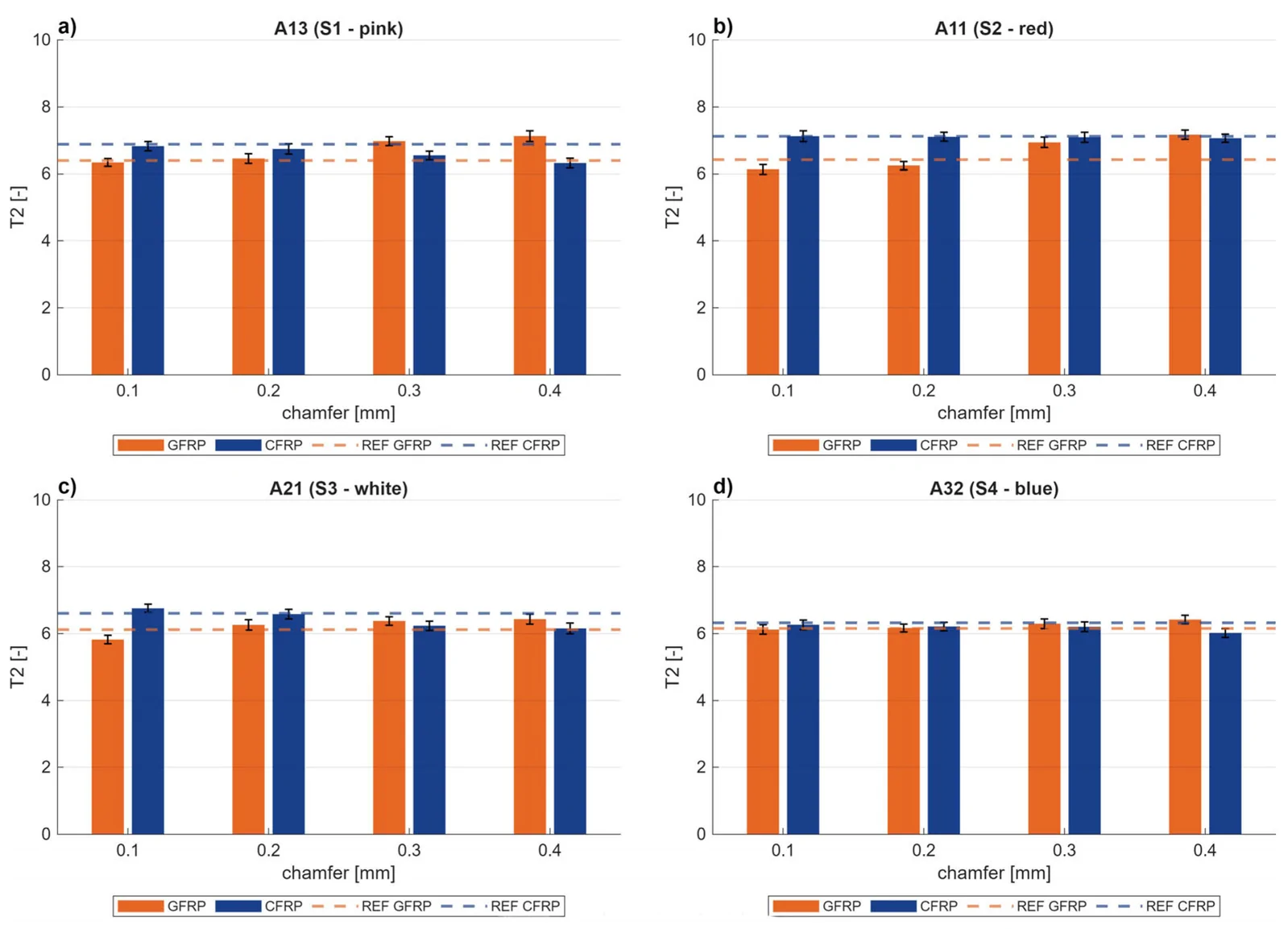
T2 as a function of chamfer depth *h* for the two materials and brushes: (**a**) S1 (A13); (**b**) S2 (A11); (**c**) S3 (A21); (**d**) S4 (A32).

Figure 14 shows the changes in T2, the recurrence time of the second type, which characterises the mean time between successive recurrence events. T2 represents the mean recurrence time between successive returns of the system to a similar state in the reconstructed phase space, excluding consecutive recurrence points belonging to the same recurrence episode. In the brushing process, T2 may reflect characteristic time scales associated with repeated fibre–workpiece interactions and the resulting force fluctuations.

The changes in T2 differed between the brushes. For brush S1 (Figure 14a), T2 increased slightly for GFRP: at 0.1 and 0.2 mm the values were close to the reference level, whereas from 0.3 mm they clearly exceeded it. For CFRP, a decreasing trend was observed: at 0.1 and 0.2 mm the values were close to the reference level, and from 0.3 mm they remained below it. For brush S2 (Figure 14b), the values for GFRP showed an increasing trend and exceeded the reference level from 0.3 mm, whereas for CFRP T2 remained practically constant and close to the reference level. For brush S3 (Figure 14c), T2 increased for GFRP: at 0.1 mm it was below the reference level, whereas from 0.2 mm it exceeded it. For CFRP, the value at 0.1 mm exceeded the reference level, at 0.2 mm it was close to it, and from 0.3 mm it remained below it. For brush S4 (Figure 14d), T2 for GFRP changed only slightly, with a small increasing tendency, and exceeded the reference level only slightly from 0.3 mm, whereas for CFRP it gradually decreased: at 0.1 and 0.2 mm the values were close to the reference level, and at greater depths they remained below it. These results indicate that T2 makes it possible to identify changes related to chamfering, but its usefulness for detecting individual chamfer depths depends on the brush and the material. The clearest deviations from the reference levels were observed for GFRP with brushes S1 and S2, and for CFRP with brushes S1 and S3.

### 3.2. Edge Condition After Brushing

**Figure 15:**
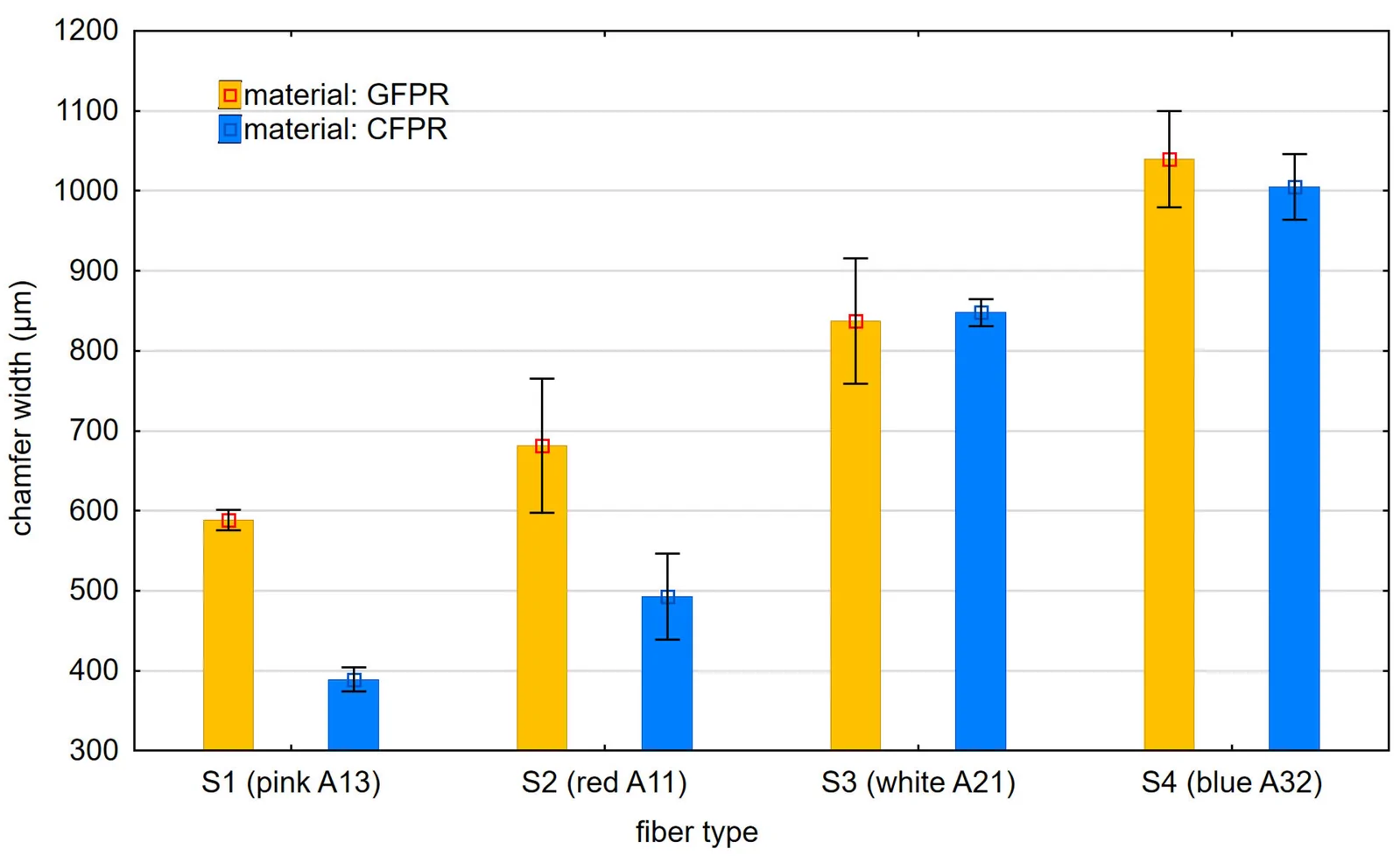
Effect of brush fibre type on the chamfer width of GFRP and CFRP edges (error bars show the standard deviation).

The edge after brushing is shown in Table 6, and the measured chamfer widths are given in Table 7 and Figure 15. All brushes removed the burrs and the sharp edge; no remaining burrs, fibre pull-out or delamination were observed under the microscope. The chamfer width increased monotonically with fibre stiffness: for GFRP from 588 ± 13 µm (S1) to 1040 ± 60 µm (S4), an increase of 77%, and for CFRP from 389 ± 15 µm to 1005 ± 58 µm, an increase of 158%. For the low-stiffness brushes (S1, S2), the chamfer on CFRP was narrower than on GFRP by 34% and 28%, respectively. For brushes S3 and S4, the differences between the materials (+1% and −3%) were within the scatter of the results. The largest relative scatter occurred for GFRP with brushes S2 and S3 (coefficient of variation approx. 12% and 9%), and the smallest for S1 (approx. 2–4% for both materials). The differences between brushes S3 and S4 (approx. 200 µm for GFRP and 160 µm for CFRP) exceed the sum of the corresponding standard deviations, which indicates a consistent trend for the tested specimens. The chamfer widths obtained (389–1040 µm) lie within the width range of the pre-chamfered segments (approx. 0.2–0.8 mm). Brushing therefore brings the edge to a condition which, particularly for the stiffer brushes, lies within the range distinguishable from the burred edge on the basis of the force signal.

The larger chamfer width on GFRP with the compliant brushes may result from differences in fibre properties and their susceptibility to microcutting, as well as from the lower coefficient of friction in contact with the carbon composite; with stiff fibres, the microcutting mechanism dominates and the effect of the material disappears. These explanations are hypotheses that were not verified by direct measurements.

## 4. Discussion

The results confirm the hypothesis that a local change in edge condition is reflected in the recurrence structure of the force signal during brushing. Under the tested conditions, the smallest chamfer distinguishable from the burred edge was approx. 0.2 mm deep (chamfer width approx. 0.4 mm, i.e., approx. 400 µm), which corresponds to 40% of the nominal depth of cut a_p_. In the RPs, this value was reached for brushes S3 and S4, while the RQA measures indicated it for most brush–material combinations. For chamfers 0.1 mm deep, the measures in most cases remained close to the reference levels, in agreement with the visual assessment of the RPs (Table 5). In milling, RQA detected defects of approximately 2% of the tool diameter [33]; the lower relative sensitivity obtained here can be explained by the brush being a tool of undefined geometry, whose force signal is the sum of the interactions of many flexible fibres randomly positioned in the contact zone [27]. For real burrs, the absolute height and the thickness of the burr root will also matter. The distinguishability of the chamfered segments obtained under the tested conditions provides a basis for developing a quantitative classification procedure, whose sensitivity and specificity will allow a detection threshold to be established in further work.

An important limitation concerns what the method actually distinguishes. The chamfered segments differ from the as-milled segments not only in the absence of burrs but also in the reduced fibre engagement (a_p_ − h), which lowers the force amplitude (Figure 7). The results therefore show that the force signal distinguishes the two edge conditions as a whole, i.e., an as-milled edge with irregular burrs and a burr-free chamfered edge, rather than the presence of individual burrs. On real edges, burrs (approx. 0.1–2 mm high in this study) also represent additional material in the contact zone, so a similar mechanism can be expected; however, this has to be verified on edges with quantified burrs and on edges actually deburred by brushing. Burr detection in the strict sense should therefore be regarded as a potential application that requires further validation. Because RR was fixed for each fragment, the RQA measures do not depend directly on the force level but on the arrangement of recurrence points.

Fibre stiffness proved to be the key factor for the visual assessment of the RPs. Low-stiffness fibres (S1) probably bend more easily, so that a local change in engagement of a few tenths of a millimetre is largely compensated by their deformation and changes the force recorded by the dynamometer only slightly. This is consistent with the intended use of these fibres, which according to the manufacturer do not change workpiece dimensions. High-stiffness fibres (S3, S4) compensate less for the change in contact conditions, which translates into more pronounced changes in the RP structure. In the RQA, the effect of fibre stiffness was less pronounced: for some measures, chamfers ≥ 0.2 mm were distinguishable also with brushes S1 and S2, suggesting that the quantitative analysis is more sensitive than the visual assessment of the RPs. Besides stiffness, the fibre types also differ in cross-sectional dimensions and shape (Table 2) and in abrasive action; the effect of stiffness therefore cannot be fully separated from these factors.

The RQA showed that the change in edge condition is reflected in the recurrence structure of the recorded signals. For chamfers ≥ 0.2 mm, DET and L mostly exceeded the reference levels of the burred edge and increased with chamfer depth, while LAM and TT mostly fell below them and decreased with chamfer depth. Higher DET and L values correspond to a larger share of diagonal structures and thus to more regular, deterministic signal dynamics on the chamfered segments, where the fibres engage less material. Lower LAM and TT values reflect fewer vertical structures and a shorter average duration of laminar states. The changes in T2 depended on the brush and the material, which limits its usefulness as a stand-alone measure.

RQA can therefore support the distinction between burred and chamfered edges. DET, L, LAM and TT, which separated consistently from the reference values, have the greatest diagnostic potential among the RQA measures analysed, and T2 can serve as a complementary measure.

The greater contrast of the RP structures for CFRP than for GFRP is the opposite of the observations for drilling, in which defects were more easily detected in GFRP [4]. A possible explanation, which requires further verification, is the different nature of the process. In brushing, the signal is generated by the contact of the fibres with the edge, and its background depends on the mode of material decohesion. Brittle carbon fibres with a high elastic modulus may produce a more uniform background signal and a sharper response to the change in edge condition.

Comparing the two parts of the study reveals a technological trade-off. Brush S4, which provides the best distinguishability in the RPs, also produces the widest chamfer (approx. 1 mm). This is advantageous when a chamfer is required by design. If only the sharp edge needs to be removed with minimal change in geometry, a less stiff brush may be preferable. For CFRP, brush S3 provides distinguishability similar to S4 (Table 5) with an approx. 16% narrower chamfer.

The results provide the basis for an adaptive deburring concept. At this stage, it is an offline proof of concept: the RPs and RQA measures were computed after the complete pass, and no real-time classifier was implemented or evaluated. If the character of the force signal on a given segment corresponds to a chamfered edge, the edge has reached the required condition, and the feed rate can be increased or an additional pass skipped. If it corresponds to a burred edge, an additional pass or a reduction in feed rate is advisable. At a constant feed rate, the time t at which a change occurs in the force signal corresponds to the position on the edge x = v_f_·t (or to the tool coordinate read from the CNC controller). Segments requiring machining can thus be located without the additional pre-process measurement needed in systems based on 3D scanning [36]. In a real-time implementation, the spatial resolution would be limited by the length of the analysed signal window and by the size of the contact zone: at v_f_ = 500 mm/min (approx. 8.3 mm/s), a window lasting 1 s corresponds to approx. 8.3 mm of the edge, to which the brush diameter (6 mm) must be added. Shorter edge segments and the transition zones between segments could not be classified reliably, and the decision would be delayed by at least the window length plus the computation time.

When interpreting these results, the conditions under which they were obtained should be taken into account. The chamfered segments differed from the as-milled segments not only in the absence of burrs but also in the reduced fibre engagement, and they were produced by milling rather than brushing; they therefore correspond to the edge condition after deburring in terms of geometry but not surface condition. Moreover, the tests were carried out for a single set of brushing parameters, a single workpiece angle, fibre extension and laminate type, and the recurrence plots were computed for the entire force signal after machining was completed. Further work will include verifying the method on edges with quantified real burrs and on edges deburred by brushing, comparing RQA with conventional force-signal statistics, assessing the effect of rotational speed, feed rate, depth of cut, edge orientation and brush wear, analogous to studies carried out for milling [33], and implementing a decision algorithm in the machine tool controller.

## 5. Conclusions

Based on the research carried out, the following conclusions were drawn:

1. The force signal recorded during edge brushing of GFRP and CFRP composites contains information about local changes in edge geometry, which can be revealed using recurrence plots.
2. Under the tested conditions, chamfered segments 0.1 mm deep were in most cases not distinguishable from the burred edge; segments 0.2–0.4 mm deep were recognisable in the RPs using brushes with high fibre stiffness (S3, S4) and, on the basis of the RQA measures, for most brush–material combinations.
3. In the RPs, distinguishability increased with fibre stiffness: the most compliant brush (S1) practically did not allow the chamfered segments on GFRP to be located, and the stiffest brush (S4) produced the most compact and legible structures.
4. For CFRP, the contrast of the RP structures was greater than for GFRP.
5. For chamfers ≥ 0.2 mm, the RQA measures DET and L mostly increased above, and LAM and TT decreased below, the reference levels of the burred edge; the changes in T2 depended on the brush and the material.
6. The chamfer width increased with fibre stiffness, from 588 to 1040 µm for GFRP and from 389 to 1005 µm for CFRP; with the compliant brushes, the chamfer on CFRP was approx. 30% narrower than on GFRP.
7. Brush selection should take into account both the required distinguishability of the edge conditions and the required edge condition; for CFRP, brush S3 provides distinguishability similar to S4 with a narrower chamfer.
8. Further research will include verifying the method on edges with quantified real burrs and on edges chamfered by brushing and assessing the effect of brushing parameters on the distinguishability of the edge conditions.

## Figures and Tables

**Table 1:** Characteristics of the composite materials tested.

Parameter	GFRP	CFRP
Resin system	916G	6376
Fabric areal weight, g/m^2^	299	285
Matrix (resin)	epoxy	epoxy
Number of plies/weave	40/8H	40/5H
Resin content, %	50	40
Manufacturing technology	autoclave	autoclave
Manufacturer/trade name	HexPly 916G/7781/50+/−2.5% (Hexcel)	HexPly 6376/40%/285-H5/T300-3K/1200 (Hexcel)

**Table 2:** Ceramic-fibre brushes used in the tests: fibre types (S1–S4), fibre cross-sections and view of the brush with the fibres extended from the sleeve.

Fibre Type	S1—A13 (Pink)	S2—A11 (Red)	S3—A21 (White)	S4—A32 (Blue)
Fibre cross-section	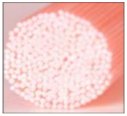	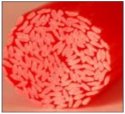	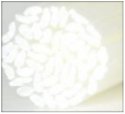	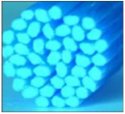
Fibre cross-section (major × minor axis), mm	0.325 × 0.325	0.690 × 0.246	1.060 × 0.433	1.112 × 0.480
Brush	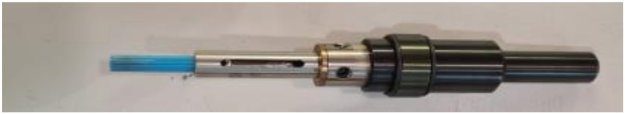			

**Table 3:** Brushing conditions.

Parameter	Value
Rotational speed *n*, rpm	5000
Feed rate *v_f_*, mm/min	500
Depth of cut *a_p_*, mm	0.5
Edge angle relative to the tool axis	45°
Brush diameter, mm	6
Fibre extension from the sleeve, mm	10
Fibre types	S1 (A13), S2 (A11), S3 (A21), S4 (A32)
Number of passes	1
Cooling	dry

**Table 4:** Recurrence quantification analysis (RQA) measures.

Measure	Symbol	Description
Recurrence rate	RR	density of recurrence points in the RP
Determinism	DET	fraction of recurrence points forming diagonal lines
Average diagonal line length	L	mean time the trajectory stays in close proximity
Laminarity	LAM	fraction of recurrence points forming vertical lines
Trapping time	TT	average length of vertical lines
Recurrence time of the second type	T2	mean time interval between successive recurrence points

**Table 5:** Visual assessment of the recognisability of chamfered segments in the RPs: − no change; ± local, ambiguous changes; + change consistent with the chamfer location; ++ distinct and compact change.

Material	Brush	*h* = 0.1 mm	*h* = 0.2 mm	*h* = 0.3 mm	*h* = 0.4 mm
GFRP	S1	−	−	−	−
	S2	−	±	+	+
	S3	−	+	++	+
	S4	−	+	++	++
CFRP	S1	−	±	±	+
	S2	−	±	+	++
	S3	−	+	++	++
	S4	±	+	++	++

Qualitative assessment based on the RPs (Figure 8 and Figure 9).

**Table 6:** Three-dimensional images of the edge after brushing with brushes S1–S4. Isolated spikes visible in the images are 3D reconstruction artefacts caused by light reflections and do not affect the chamfer width measurement.

Material	S1—A13 (Pink)	S2—A11 (Red)	S3—A21 (White)	S4—A32 (Blue)
GFRP	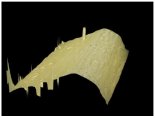	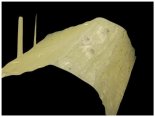	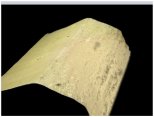	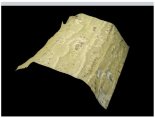
CFRP	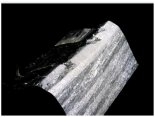	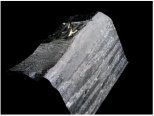	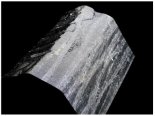	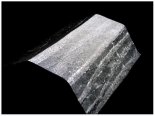

**Table 7:** Chamfer width b_f_ on the reference segments (without a pre-chamfer) after brushing (mean ± standard deviation, n = 5).

Brush	GFRP b_f_, µm	CFRP b_f_, µm	Difference CFRP vs. GFRP
S1 (pink)	588 ± 13	389 ± 15	−34%
S2 (red)	681 ± 84	493 ± 54	−28%
S3 (white)	837 ± 78	848 ± 17	+1%
S4 (blue)	1040 ± 60	1005 ± 58	−3%

## Data Availability

The data presented in this study are available on request from the corresponding author.
